# Effect of alginate coatings incorporated with chitinase from ʻBaozhuʼ pear on the preservation of cherry tomato during refrigerated storage

**DOI:** 10.1002/fsn3.2908

**Published:** 2022-04-27

**Authors:** Yongmin Wu, Yi Wu, Peng Han, Jiangqi Xu, Xiaobo Liang

**Affiliations:** ^1^ 47910 Faculty of Food Science and Engineering Kunming University of Science and Technology Kunming China; ^2^ 58276 Beijing Key Laboratory of Flavor Chemistry School of Light Industry Beijing Technology and Business University Beijing China

**Keywords:** ʻBaozhuʼ pear chitinase, cherry tomatoes, edible coatings, *Fusarium oxysporum*, sodium alginate

## Abstract

The effects of edible coatings based on sodium alginate with ʻBaozhuʼ pear chitinase on the quality of cherry tomatoes during refrigerated storage were evaluated. Cherry tomatoes inoculated with *Fusarium oxysporum* were coated and stored up to 21 days. All coatings with the chitinase significantly reduced *F*. *oxysporum* proliferation on cherry tomatoes during storage and extended the shelf life of cherry tomatoes effectively (*p* < .05). Results showed that alginate coatings with the chitinase could prevent weight loss, maintain firmness, and slow down the changes of titratable acidity and vitamin C (*p* < .05) in a dose‐dependent manner. However, no significant differences were observed between T3 (1% alginate/0.15% ʻBaozhuʼ pear chitinase/1% glycerin) and T4 (1% sodium alginate/0.3% ʻBaozhuʼ pear chitinase/1% glycerin) (*p* > .05). Overall, alginate coating with 0.15% ʻBaozhuʼ pear chitinase could be a promising method to maintain the quality of cherry tomatoes.

## Graphical abstract

Chitinase is first used in edible coating. This report focus on problem of Fusarium oxysporum on cherry tomato.

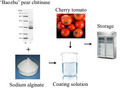

## 1 INTRODUCTION

Cherry tomato (*Lycopersicon esculentum*) is one of the most popular fruits worldwide due to its high content of vitamin C and β‐carotene. However, it is a climacteric fruit and highly perishable (Su et al., 2021). The main factors that affect its quality are ethylene production, the environment, and deterioration caused by fungi. The phytopathogen *Fusarium oxysporum* causes nearly 60% of production loss of tomato (Medina‐Romero et al., 2017).

In the last decades, the increasing demand for fresh fruit and vegetable, without any synthetic preservatives, has driven requirements for natural alternatives that prolong fruit shelf life (da Costa de Quadros et al., 2020). Edible coating combined with bioactive compound was considered as an excellent solution (Oyom et al., 2022; Riaz et al., 2021). Coatings with ethanol extract of propolis and fish protein hydrolysate can prevent contamination by fungi such as *Penicillium chrysogenum*, *Fusarium solani*, and *Botrytis cinerea* in cherry tomato (da Costa de Quadros et al., 2020; Pobiega et al., 2020). However, no study has attempted to control *F*. *oxysporum* in cherry tomato on the basis of edible coating.

ʻBaozhuʼ pear (*Pyrus ussuriensis* Maxim) is one of the popular fruits in China. It is mainly produced in Yunnan Province, and this pear shows great resistance to fungal diseases. Our group found that the chitinase from ʻBaozhuʼ pear was probably the reason for its fungal resistance. The chitinase exhibits antifungal activity toward *Trichoderma viride*, *F*. *solani*, *Rhizoctonia solani*, and especially *F*. *oxysporum* (Han et al., 2016). While alginate coatings have been established for the potential preservation of fruits and vegetables, including cherry tomato (Nair et al., 2020; Zhu et al., 2019), no study has used antifungal chitinase in edible coating for food preservation to date. The aim of this study was to evaluate the effects of alginate coatings incorporated with chitinase from ʻBaozhuʼ pear on the quality of cherry tomatoes during refrigerated storage.

## 2 MATERIALS AND METHODS

### 2.1 Materials

ʻBaozhuʼ pear and cherry tomato (*Lycopersicon esculentum Mill*. cv. ‘Mali’) was obtained from the local market. The ʻBaozhuʼ pear and cherry tomatoes were picked at ripening and turning stage (red color covering between 60% and 90% of fruit surface), respectively, and transported to the laboratory immediately after harvesting. The fruits were selected according to uniform color, size, shape, and the absence of damage and fungal infection. *F*. *oxysporum* was bought from Microbial Culture Collection Center of Guangdong Institute of Microbiology, Guangdong, China. Sodium alginate (molecular weight: 216) was purchased from Aladdin Biochemical Technology Co., Ltd, Shanghai, China. All other chemicals and reagents used were of analytical grade.

### 2.2 Preparation of chitinase

Chitinase was prepared according to method of Han et al. (2016). The juice of ʻBaozhuʼ pear was subjected to 40%–80% saturation of ammonium sulfate. The protein was dialyzed by 20 mM phosphate buffer (pH 7.4) and went through a 0.22 μm filter.

The chitinase activity was checked according to the method of Han et al. (2016). Protein concentration was measured according to Bradford method (Bradford, 1976).

### 2.3 Preparation and application of coating treatments to cherry tomatoes

Coating solutions were prepared according to the method of Zhu et al. (2019) with some modification. The sodium alginate solution was prepared by mixing a certain 1% (w/v) sodium alginate and 1% (w/v) glycerin in distilled water. Further, different concentrations (0%, 0.075%, 0.15%, 0.3%) of ʻBaozhuʼ pear chitinase were added to each of the sodium alginate solutions. The final coatings were listed in Table 1.

**TABLE 1:** Coating treatments to cherry tomatoes

Treatment	Coatings
T1	1% sodium alginate/0% ʻBaozhuʼ pear chitinase/1% glycerin
T2	1% sodium alginate/0.075% ʻBaozhuʼ pear chitinase/1% glycerin
T3	1% sodium alginate/0.15% ʻBaozhuʼ pear chitinase/1% glycerin
T4	1% sodium alginate/0.3% ʻBaozhuʼ pear chitinase/1% glycerin

The cherry tomatoes were randomly separated into five groups. First of all, cherry tomatoes were selected, randomized, washed with a fruit detergent, rinsed with tap water, and allowed to air‐dry at room temperature. Then samples were wounded once in the equator with a stainless steel rod with a probe tip 1 mm wide and 2 mm in length. This wound was inoculated with the pathogen by placing 10 μl of a spore suspension containing 1 × 10^6^ spores/ml of *F*. *oxysporum*. After incubation at 20°C for 24 h, inoculated fruit were coated by immersion for 30 s in the coating solutions, drained, and allowed to air‐dry at 20°C. Inoculated but uncoated samples were used as control (CK). All samples were placed in the fruit packing box and stored for 21 days during refrigerated storage at 55%–60% RH. Samples were randomly taken out and analyzed at intervals of 3 days.

### 2.4 Effect of coatings on cherry tomatoes quality

#### 2.4.1 Mold count

Mold count of cherry tomato was conducted according to the method of da Costa de Quadros et al. (2020). Samples (30 g) were homogenized in 270 ml of sterile peptone water (0.1%; w/v) by Stomacher blender (Zhixin, China). The homogenized samples were diluted properly and inoculated on potato dextrose agar. The plates inoculated were held at 25°C for 5 days. Counts were expressed as log CFU/g in triplicate.

#### 2.4.2 Weight loss

The weight loss was determined as described by AOAC (2000). Ten samples of each treatment were weighed nondestructively. The weight loss was expressed as a percentage in relation to the initial weight.

#### 2.4.3 Firmness

Firmness analysis was performed on cherry tomato using a TA‐XT plus texture analyzer (Stable Micro System, UK). The test was performed twice at an interval of 0 s at 25% compression; the P75 cylindrical probe moved at a constant speed of 1.5 mm/s. Six cherry tomatoes were used per replicate.

#### 2.4.4 Chemical properties

Titratable acidity (TA) was measured according to method of Won et al. (2018). In brief, ten samples of each treatment were taken out every 3 days. NaOH (0.1 mol/L) was used to titrate until the pH of diluted juice (5 ml) reached 8.1. TA was represented as a percentage.

Vitamin C content was determined by the 2,6‐dichloroindophenol titrimetric method (Rashida et al., 1997). In brief, a 30 g homogenized sample was blended with about 100 ml of 2% oxalic acid. The blended mixture was made to 500 ml with 2% oxalic acid and was filtered; 10 ml of the filtrate were titrated with standard 2,6‐dichloroindophenol. Results were expressed as mg per 100 g wet basis.

#### 2.4.5 Sensory evaluation

Sensory analysis was evaluated by the method of Won et al. (2018) with some modifications. Glossiness, color, texture, and overall acceptability were used as assessed terms. Six trained members from the Faculty of Food Science and Engineering at Kunming University of Science and Technology took participated in the evaluation. The criteria were designed in 9‐point scale (1 = disliked extremely, 5 = neither liked nor disliked, and 9 = liked extremely).

### 2.5 Statistical analysis

Statistical analysis was performed by the method of Li et al. (2018). One‐way analysis of variance (ANOVA) and Pearson's regression were employed for paired comparison and correlation, respectively, using Origin version 9.0.

## 3 RESULTS

### 3.1 Mold count

The changes in mold count are shown in Table 2. The results showed that the cherry tomatoes covered with alginate coatings incorporating chitinase were better during refrigerated storage compared with those without the chitinase (T1 and CK). The initial values were approximately 4.3 log_10_ CFU/g for all samples. An increasing trend was observed for all samples. After 6 days of storage, obvious differences were observed between the groups with relatively high concentration of chitinase (T3, T4) and without chitinase (CK, T1). Moreover, there were no significant differences between T1 and CK, and no significant differences were observed between T3 and T4 (*p* > .05). Among groups with chitinase, an obvious difference was observed between T2 and T3 and T4 on the 9th–15th day of storage. On the 15th day, T1 and control groups were spoiled (mold visible), and the mold count in the control group exceeded 6.5 log_10_ CFU/g. In addition, the mold count increased faster among groups with chitinase, especially under relatively high concentration after the 15th day of storage. At the end of storage time, the mold counts among groups with chitinase were almost same.

**TABLE 2:** Mold counts of cherry tomatoes during refrigerated storage

Parameter	Storage time (d)	CK	T1	T2	T3	T4
Mold count (CFU/g)	0	4.28 ± 0.10^Aa^	4.25 ± 0.20^Aa^	4.27 ± 0.13^Aa^	4.26 ± 0.09^Aa^	4.27 ± 0.10^Aa^
	3	4.96 ± 0.07^Ba^	4.90 ± 0.05^Bab^	4.75 ± 0.12^Bbc^	4.66 ± 0.06^Bc^	4.55 ± 0.06^Bc^
	6	5.42 ± 0.12^Ca^	5.24 ± 0.09^BCab^	5.03 ± 0.11^Bbc^	4.88 ± 0.12^BCc^	4.75 ± 0.17^BCc^
	9	5.77 ± 0.11 Da	5.58 ± 0.06^Cab^	5.41 ± 0.05^Cb^	5.06 ± 0.13^Cc^	4.93 ± 0.11^CDc^
	12	6.17 ± 0.04^Ea^	6.04 ± 0.03 Da	5.74 ± 0.03^Db^	5.33 ± 0.06^Dc^	5.14 ± 0.15^Dc^
	15	6.53 ± 0.05^Fa^	6.32 ± 0.10^Db^	6.00 ± 0.07^Dc^	5.71 ± 0.04^Ed^	5.49 ± 0.08^Ee^
	18	6.91 ± 0.08 Ga	6.77 ± 0.07^Ea^	6.34 ± 0.12^Eb^	6.22 ± 0.11^Fbc^	6.03 ± 0.14^Fc^
	21	7.73 ± 0.05^Ha^	7.52 ± 0.23^Fa^	6.87 ± 0.14^Fb^	6.68 ± 0.03^Gb^	6.55 ± 0.04^Gb^

Note

All values are the mean ± standard deviation (*n* = 3).

A–H means with different letters within the same treatments are significantly different (*p* < .05).

a–c means with different letters within the same day of storage time are significantly different (*p* < .05).

### 3.2 Weight loss

**FIGURE 1:**
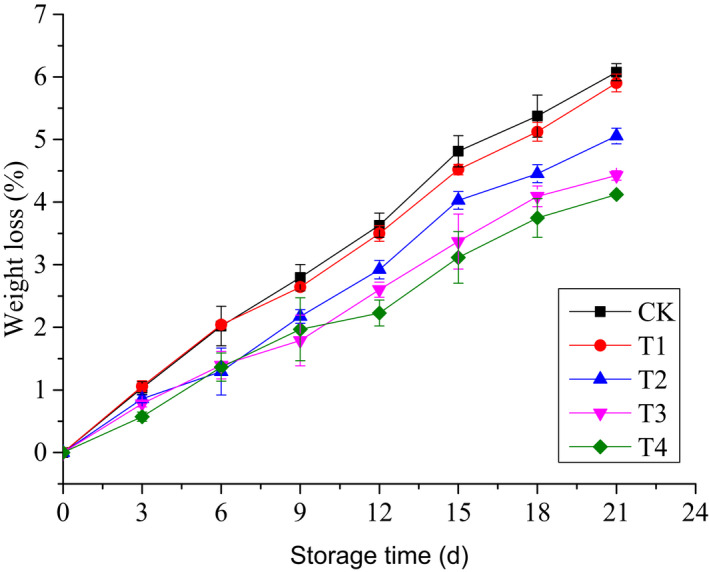
Changes in weight loss of cherry tomatoes treated by edible coatings based on sodium alginate with ʻBaozhuʼ pear chitinase during refrigerated storage

The weight loss from cherry tomatoes during refrigerated storage is shown in Figure 1. A similar increasing trend can be observed for all samples. The values of groups incorporated with chitinase showed a prominent decrease compared to the control and T1 groups, and the effect was better when the concentration of chitinase was increased. Furthermore, no significant differences were observed between T1 and CK, and T3 and T4 showed similar weight loss reduction. T3 showed significant prevention on weight loss (4.43%) compared to the CK (6.08%) at the end of storage time (*p* < .05).

### 3.3 Firmness

**FIGURE 2:**
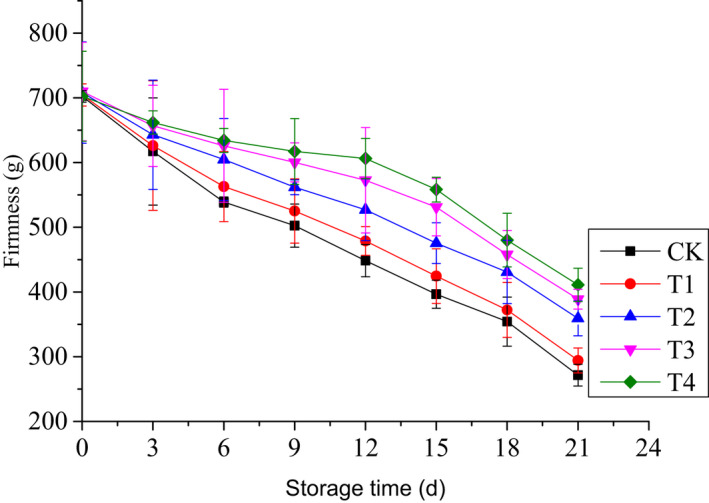
Changes in firmness of cherry tomatoes treated by edible coatings based on sodium alginate with ʻBaozhuʼ pear chitinase during refrigerated storage

Figure 2 shows the firmness changes of cherry tomatoes during refrigerated storage. The firmness values gradually decreased during storage. Treatment T4 resulted in a higher reduction of fruit firmness compared with all other treatments. After 6 days of storage, there were significant differences between groups with relatively high concentration of chitinase (T3, T4) and without chitinase (CK, T1) (*p* < .05). In terms of mold count, significant differences were observed between T2 and T3 and T4 on 9th–15th day of storage. As shown in Figure 2, the value of T4 was higher than those of other groups. However, T4 had no significant difference with T3 (*p* > .05), and no significant differences were observed between T1 and CK (*p* > .05).

### 3.4 Titratable acidity

The TA of cherry tomatoes during storage is shown in Table 3, which presented a downward trend during storage for all samples. After 21 days of storage, the TA contents in cherry tomatoes were significantly lower than the initial values (*p* < .05). Alginate coatings incorporating chitinase could result in a lower reduction of TA compared to those without chitinase (*p* < .05). However, no significant differences were observed among groups with chitinase and groups without chitinase (*p* > .05). After 21 days of storage, the TA content of the CK group was only 0.13%, which decreased by 70.5%, whereas that of the T4 group was 0.24%, which decreased by 44.2%.

**TABLE 3:** Titratable acidity of cherry tomatoes during refrigerated storage

Parameter	Storage time (d)	CK	T1	T2	T3	T4
Titratable acidity (%)	0	0.44 ± 0.03^Aa^	0.43 ± 0.02^Aa^	0.41 ± 0.02^Aa^	0.42 ± 0.02^Aa^	0.43 ± 0.03^Aa^
	3	0.36 ± 0.02^Ba^	0.38 ± 0.01^Ba^	0.38 ± 0.01^ABa^	0.38 ± 0.01^ABa^	0.39 ± 0.01^ABa^
	6	0.31 ± 0.01^BCa^	0.33 ± 0.02^BCab^	0.35 ± 0.02^ABCab^	0.37 ± 0.03^ABCb^	0.38 ± 0.03^ABCb^
	9	0.28 ± 0.02^Ca^	0.30 ± 0.01^CDab^	0.33 ± 0.02^BCDbc^	0.34 ± 0.01^BCDc^	0.35 ± 0.01^BCDc^
	12	0.25 ± 0.03^CDa^	0.27 ± 0.03^DEa^	0.29 ± 0.06^CDEa^	0.32 ± 0.03^CDEa^	0.33 ± 0.01^CDEa^
	15	0.21 ± 0.04^DEa^	0.23 ± 0.02^EFab^	0.26 ± 0.01^DEFabc^	0.29 ± 0.02^DEbc^	0.32 ± 0.03^DEc^
	18	0.18 ± 0.01^EFa^	0.20 ± 0.03^FGab^	0.23 ± 0.03^EFabc^	0.27 ± 0.02^EFbc^	0.28 ± 0.03^EFc^
	21	0.13 ± 0.02^Fa^	0.15 ± 0.01 Ga	0.20 ± 0.02^Fb^	0.22 ± 0.03^Fb^	0.24 ± 0.01^Fb^

Note

All values are the mean ± standard deviation (*n* = 3).

A–G means with different letters within the same treatments are significantly different (*p* < .05).

a–c means with different letters within the same day of storage time are significantly different (*p* < .05).

### 3.5 Vitamin C

**FIGURE 3:**
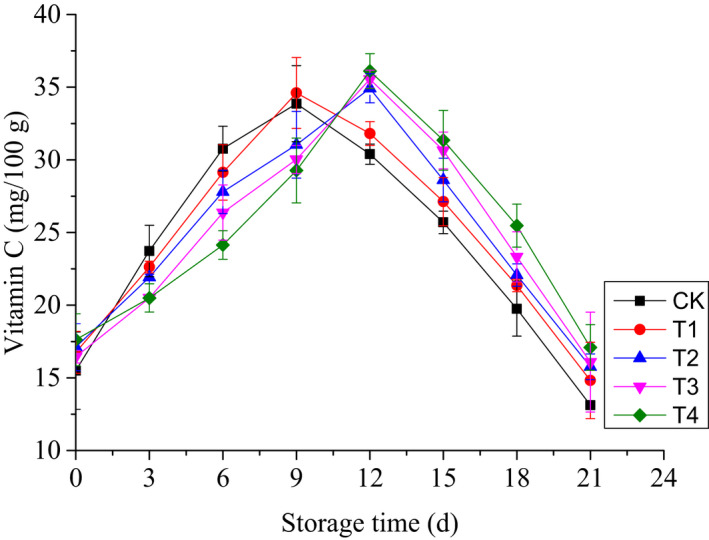
Changes in vitamin C of cherry tomatoes treated by edible coatings based on sodium alginate with ʻBaozhuʼ pear chitinase during refrigerated storage

Vitamin C content in cherry tomatoes increased initially and then decreased with time (Figure 3). The initial vitamin C content of cherry tomatoes ranged from 15.5 mg/100 g to 17.6 mg/100 g. On the ninth day, the content in CK and in T1 groups reached a peak value of 34.0 mg/100 g. However, compared to the control and T1 groups, the peak value for groups with chitinase was observed 3 days later, and the values were approximately 35.5 mg/100 g. Furthermore, significant differences were observed between the groups with and without chitinase (*p* < .05). However, similar to the TA content, no significant differences were observed among groups with and without chitinase (*p* > .05).

### 3.6 Sensory properties

The changes in sensory properties are shown in Table 4. The results showed that the sensory properties of cherry tomatoes changed significantly (*p* < .05) and improved by alginate coatings, especially with chitinase (T2, T3, and T4). The control and T1 groups had a shelf life of 12 days, and groups with chitinase had an extended shelf life of 3 days. The values presented a similar trend that decreasing after increasing in the beginning except for glossiness with decreasing trend. The results showed that there was no statistically significant difference in terms of glossiness, color, texture, and overall acceptability among groups with and without chitinase (*p* > .05).

**TABLE 4:** Sensory evaluation of cherry tomatoes during refrigerated storage

Sensory index	Storage time (d)	Control	T1	T2	T3	T4
Glossiness	0	8.8 ± 0.4^Aa^	8.7 ± 0.5^Aa^	8.7 ± 0.5^Aa^	8.7 ± 0.5^Aa^	8.7 ± 0.5^Aa^
	3	8.7 ± 0.5^Aa^	8.5 ± 0.5^Aa^	8.8 ± 0.4^Aa^	8.8 ± 0.4^Aa^	8.8 ± 0.4^Aa^
	6	7.7 ± 0.5^Ba^	8.2 ± 0.4^Aa^	8.5 ± 0.5^Aa^	8.5 ± 0.5^Aa^	8.5 ± 0.5^Aa^
	9	6.7 ± 0.5^Ca^	7.8 ± 0.4^Ab^	8.2 ± 0.4^Ab^	8.2 ± 0.4^Ab^	8.3 ± 0.5^Ab^
	12	5.5 ± 0.5 Da	6.5 ± 0.5^Bb^	6.7 ± 0.5^Bb^	6.8 ± 0.4^Bb^	7.0 ± 0.6^Bb^
	15	4.3 ± 0.5^Ea^	4.7 ± 0.5^Cab^	5.3 ± 0.5^Cbc^	5.5 ± 0.5^Cbc^	5.7 ± 0.5^Cc^
	18	3.3 ± 0.5^Fa^	4.2 ± 0.4^CDb^	4.0 ± 0.6^Dab^	4.5 ± 0.5^Db^	4.5 ± 0.5^Db^
	21	3.2 ± 0.4^Fa^	3.3 ± 0.5 Da	3.8 ± 0.8 Da	3.8 ± 0.4 Da	4.2 ± 0.4^Db^
Color	0	6.8 ± 0.4^ACa^	6.7 ± 0.5^Aa^	6.7 ± 0.5^Aa^	6.7 ± 0.5^Aa^	6.7 ± 0.5^Aa^
	3	7.2 ± 0.4^ACa^	7.2 ± 0.4^Aa^	7.2 ± 0.4^ACa^	7.2 ± 0.4^ACa^	7.2 ± 0.4^ACa^
	6	8.2 ± 0.4^Ba^	7.5 ± 0.4^ABab^	7.3 ± 0.5^ABCb^	7.3 ± 0.5^ABCb^	7.3 ± 0.5^ABCb^
	9	7.7 ± 0.5^ABa^	8.2 ± 0.4^Ba^	7.7 ± 0.5^BCa^	7.7 ± 0.5^BCa^	7.7 ± 0.5^BCa^
	12	6.3 ± 0.5^Ca^	7.2 ± 0.4^Ab^	7.8 ± 0.4^Cab^	7.8 ± 0.4^Cb^	7.8 ± 0.4^Cb^
	15	4.7 ± 0.5 Da	4.8 ± 0.8^Ca^	5.7 ± 0.5^Db^	5.7 ± 0.5^Db^	5.8 ± 0.4^Db^
	18	3.7 ± 0.5^Ea^	4.3 ± 0.5^CDa^	4.3 ± 0.5^Ea^	4.5 ± 0.5^Ea^	4.5 ± 0.5^Ea^
	21	3.7 ± 0.5^Ea^	3.7 ± 0.5 Da	3.8 ± 0.4^Ea^	4.0 ± 0.6^Ea^	3.8 ± 0.4^Ea^
Texture	0	5.6 ± 0.5^ACa^	5.6 ± 0.5^ACa^	5.6 ± 0.5^ADa^	5.6 ± 0.5^ACa^	5.6 ± 0.5^ACa^
	3	6.2 ± 0.4^ACa^	6.2 ± 0.4^ABCa^	6.2 ± 0.4^ABDa^	6.2 ± 0.4^ABCa^	6.2 ± 0.4^ABCa^
	6	7.5 ± 0.5^Ba^	6.8 ± 0.4^Bab^	6.7 ± 0.5^BCab^	6.5 ± 0.5^ABb^	6.5 ± 0.5^ABCb^
	9	6.7 ± 0.5^ABa^	6.7 ± 0.5^BCa^	7.2 ± 0.4^Ca^	6.8 ± 0.4^Ba^	6.8 ± 0.4^BCa^
	12	5.5 ± 0.5^Ca^	5.8 ± 0.4^Ca^	6.3 ± 0.5^ACab^	6.7 ± 0.5^Bb^	7.2 ± 0.4^Bb^
	15	4.2 ± 0.4 Da	4.6 ± 0.5^Dab^	5.3 ± 0.5^DEbc^	5.5 ± 0.5^Cc^	5.8 ± 0.8^Cc^
	18	3.5 ± 0.5 Da	4.5 ± 0.5^Db^	4.5 ± 0.5^EFb^	4.3 ± 0.5^Dab^	4.2 ± 0.8^Dab^
	21	3.3 ± 0.8 Da	3.5 ± 0.5^Ea^	3.7 ± 0.5^Fa^	3.7 ± 0.5 Da	3.7 ± 0.5 Da
Overall	0	6.7 ± 0.5^Aa^	6.7 ± 0.5^ACa^	6.7 ± 0.5^Aa^	6.7 ± 0.5^Aa^	6.7 ± 0.5^ABa^
	3	7.0 ± 0.6^ABa^	7.2 ± 0.4^ABCa^	7.0 ± 0.6^Aa^	7.2 ± 0.4^Aa^	7.2 ± 0.4^Aa^
	6	7.7 ± 0.5^Ba^	7.5 ± 0.5^ABa^	7.3 ± 0.5^Aa^	7.5 ± 0.5^Aa^	7.5 ± 0.5^Aa^
	9	6.5 ± 0.5^Aa^	7.2 ± 0.4^Bab^	7.5 ± 0.5^Ab^	7.5 ± 0.5^Ab^	7.5 ± 0.5^Ab^
	12	5.5 ± 0.5^Ca^	6.2 ± 0.4^Cab^	6.7 ± 0.5^Ab^	6.8 ± 0.4^Ab^	6.2 ± 0.4^Bab^
	15	4.2 ± 0.4 Da	4.7 ± 0.5^Dab^	5.3 ± 0.5^Bbc^	5.7 ± 0.5^Bc^	5.8 ± 0.4^Bc^
	18	3.3 ± 0.5^DEa^	4.2 ± 0.4^Db^	4.2 ± 0.4^Cb^	4.3 ± 0.5^Cb^	4.5 ± 0.5^Cb^
	21	2.8 ± 0.4^Ea^	3.2 ± 0.4^Eab^	3.7 ± 0.5^Cbc^	3.8 ± 0.4^Cbc^	4.2 ± 0.4^Cc^

Note

All values are the mean ± standard deviation (*n* = 6).

A–D means with different letters within the same treatments are significantly different (*p* < .05).

a–b means with different letters within the same day of storage time are significantly different (*p* < .05).

## 4 DISCUSSION

Postharvest fruits are easy to rot because of phytopathogen fungi (Cortés et al., 2020; Di Liberto et al., 2020). Biological coating has been proven to be a safe and effective way to maintain fruit quality by preventing fungal invasion (Garcia et al., 2021; Hassan et al., 2020). In the present study, alginate coatings with ʻBaozhuʼ pear chitinase had a positive effect on the quality of cherry tomato inoculated with *F*. *oxysporum* during refrigerated storage.

The results showed that groups with ʻBaozhuʼ pear chitinase could efficiently inhibit the increasing mold count. At the same time, no significant difference was observed between T1 and CK (*p* > .05), which was different from previous reports (Liu et al., 2021; Silva et al., 2021; Tabassum et al., 2020). This result was probably because the alginate coating was broken by fungi. The effect on controlling mold improved when the concentration of chitinase was increased. However, no significant difference was observed between T3 and T4, which was probably due to the limit of the antifungal activity of chitinase (Han et al., 2016). Moreover, the values of mold count increased faster among groups with relatively high concentration of chitinase after the 15th day of storage, which was likely because the chitinase was unstable during long storage time and became nutriment to the fungi.

Among physicochemical parameters, weight loss is one of the crucial factors affecting the commercial value of fruit (Ktenioudaki et al., 2021). Weight changes are mainly due to water transpiration of the fruit (Aparicio‐García et al., 2021). The results revealed that alginate coatings with chitinase could prominently decrease the weight loss and the effect was concentration dependent, which was likely attributed to the fact that chitinase could maintain the integrity of alginate coating. However, no significant difference was observed between T3 and T4 (*p* > .05), which was correlated with the variations of mold count (*p* < .05). This was different from the result obtained in a previous study wherein alginate coating slowed down weight loss obviously (Duong et al., 2022; Liu et al., 2021; Silva et al., 2021). No significant difference was observed between T1 and CK, which was likely due to alginate coating being broken by fungi. The firmness values were significantly correlated with weight loss values (*p* < .05), which is similar to previous reports (da Costa de Quadros et al., 2020; Yoo et al., 2021). These results showed that the lesser the weight loss, the greater the firmness of cherry tomato.

The reason for the decreasing TA content was that acids are the main substrates of respiratory metabolism (Xing et al., 2021; Yang et al., 2021). Alginate coating could delay the changes in acidity by reducing the respiration rate (Carbone et al., 2021; Zhang et al., 2017). As shown in Figure 3, T1 (1% sodium alginate/1% glycerin) had no beneficial effect on TA compared to the control samples, which might also be attributed to alginate coating being broken by fungi. The existence of chitinase could maintain the integrity of alginate coating, indicating the alginate coatings with ʻBaozhuʼ pear chitinase had good effect on TA. Furthermore, the vitamin C content increased initially and then decreased with time, and changes in vitamin C content were delayed as the coatings with chitinase. The amount of ascorbic acid is formed at the pink stage and then decreases at the red stage of tomato ripening. However, the decline of vitamin C content in fruit results from respiration and oxidation (Kaewklin et al., 2018; Mieszczakowska‐Frąc et al., 2021; Wu et al., 2016), which could be the reasons for its variation.

Sensory evaluation showed that alginate coatings with chitinase could efficiently extend the shelf life of cherry tomatoes inoculated with *F*. *oxysporum* (*p* < .05). According to the mold count, the limit of value is around 6.5 log_10_ CFU/g. These results indicated that edible coating with antifungal chitinase is an effective way to prevent fungal contamination in fruits.

## 5 CONCLUSIONS

This study showed that alginate coatings with the ʻBaozhuʼ pear chitinase were able to inhibit the proliferation of *F*. *oxysporum* and extend the shelf life of cherry tomato. Moreover, the coating containing the chitinase significantly improved the physicochemical and sensory properties of cherry tomatoes during refrigerated storage (*p* < .05). Nevertheless, no significant differences were observed between T3 and T4 (*p* > .05). Thus, T3 (1% alginate/0.15% ʻBaozhuʼ pear chitinase/1% glycerin) could maintain the quality of cherry tomato.

## CONFLICT OF INTEREST

The authors have declared no conflicts of interest for this article.

## Data Availability

Data available on request from the authors.
